# Generation of Low-Cost User-Customizable Neutral Density Filters and the Involvement of Undergraduate Researchers

**DOI:** 10.1021/acsomega.5c07533

**Published:** 2025-12-19

**Authors:** Julia Filip, Joseph Eppich, Melissa Y. Gallardo, Andrew S. Hamilton, Noelle Vermost, Kevin W. Davies

**Affiliations:** Department of Chemistry and Physics, Florida Gulf Coast University, 10501 FGCU Blvd South, Fort Myers, Florida 33965-6565, United States

## Abstract

In research and teaching laboratories, optical attenuation of a light source is often required on short notice, yet access to suitable filters may be limited, resulting in delays or increased expense. For monochromatic applications, attenuation can be achieved using individual solutions of absorptive dyes. However, broadband or spectral work typically necessitates a suite of neutral density (ND) filters covering the desired wavelength range and attenuation levels. This work presents a low-cost, customizable approach for formulating liquid ND filters from common laboratory dyes. Two scenarios are considered: laboratories sourcing dyes for purchase and those leveraging existing dye inventories. An example ND solution prepared from in-house materials rivals commercial ND filters: from 425 to 575 nm, abs. range 2.04–2.16 (0.7–0.9%T), abs_avg_ = 2.09 (0.8%T), and s.d._abs_ = 0.03. This stock solution can be diluted as needed and is compatible with a range of optical systems (e.g., OPO lasers, fluorescence setups). The formulation strategy is generalizable to other dye sets and application requirements.

## Graphical abstract

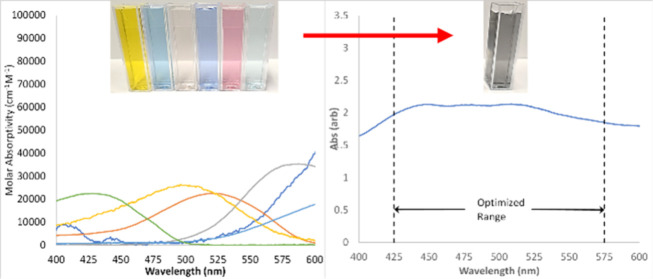

## Introduction

Neutral density (ND) filters are frequently used in both laboratory research and teaching environments to attenuate light intensity to protect sensitive detectors or prevent signal saturation (e.g., fluorescence measurements on instruments without user-controllable amplification). For single-wavelength applications, attenuation can be economically achieved using dye solutions as attenuators diluted to a desired transmittance, and placed within the optical path in a mounted cuvette. This method is simple, reproducible, and effective for many routine setups, and has been employed in various photoacoustic studies.^1,2^ While thermal lensing may arise at high optical intensities,^3^ it is generally not a limiting factor for typical experimental conditions encountered in research or teaching environments.

However, for spectral work this approach becomes prohibitively complex and cumbersome, as each wavelength requires a separate attenuation solution. Instead, commercially available ND filters are generally used, which uniformly absorb across a broad spectral range. ND filters of varying absorbances, wavelength coverage, and attenuation mechanisms (e.g., reflective, absorptive, polarizing) are commercially available and perform reliably in stable instrumental configurations. In novel, custom, or temporary setups, however, the filters themselves and the necessary optomechanical hardware can introduce considerable cost and delay (e.g., ordering, shipping, integration).

To address this, we developed a customizable (abs, λ range, flatness, etc.) filter design spreadsheet that, once populated with the absorbance spectra of already-owned dyes (or dyes under consideration for purchase), can be used to design a combination of dyes/concentrations that will yield a satisfactory ND filter. The resulting solution is shelf stable, compatible with high intensity light sources, dilutable on demand to the desired attenuation level, and cost-effective. We further compare this liquid-based attenuation method with traditional solid state ND filters, evaluating limitations and application contexts for each.

We consider two practically relevant and potentially overlapping use cases. First, in the “clean start” scenario, a lab begins with no suitable dyes on hand and designs the filter entirely from published absorbance spectra to guide future dye purchases. Second, in the “existing supply” scenario, a lab leverages absorbance spectra from dyes already in inventory. To evaluate both pathways, we designed and compared ND filter solutions using each approach. These results demonstrate that high-quality, low-cost ND filters can be formulated using either in-house spectral data or processed external sources.

### Undergraduate Research: Turning Difficulties into Opportunities

This project originated during the early stages of the COVID-19 pandemic, shortly before in-person activities were curtailed. This presented a difficult challenge: how could students enrolled for research credit engage in meaningful scientific work without access to a laboratory? Additionally, how could a faculty member structure such a remote experience in a way that also advanced their own research goals? In response, we developed a hybrid strategy that enabled undergraduate students to work remotely using literature-based spectral data and computational modeling. This approach not only ensured continuity in student research but also directly shaped the technical development of the ND filter system described herein. The educational dimension of this work is revisited in the Discussion section, where we reflect on its broader pedagogical implications.

## Experimental Section

### Initial Dye Set Selection

In selecting our candidate dyes, our goal was to select dyes that contributed major absorbance peaks over various portions of the wavelength range. Absorbance peak height was not a primary concern at this stage, as final attenuation would be controlled through concentration optimization. We model two scenarios: the ‘Existing Supply’ case, in which a lab designs an ND filter from in-house dyes, and the ‘Clean Start’ case, in which a lab selects dyes for purchase. We selected 16 dyes already present in our inventory to model both approaches.

Our selection emphasized low-cost dyes with absorbance peaks distributed across the wavelength range of interest (in this example 425–575 nm); to ensure spectral coverage, we ensured that dyes had significant peaks in one of three ‘zones’ of our desired spectral range. It is important to have a variety of dyes with their core peak in these ranges, as the fitting approach will also need to contend with a dye’s additional peaks. These dye ranges deliberately extend beyond the wavelength range to be optimized, ensuring the dyes selected will maintain flatness to the edges of this range.

The absorptivity spectra of these initial dyes are shown in Figure 1. In the “existing supply” scenario, spectra were measured in-house under known concentration conditions, allowing for direct conversion to molar absorptivity, ε (cm^–1^ M^–1^), using a Shimadzu UV-2450 scanning double beam UV–vis spectrophotometer. In the “clean start” scenario, spectra were sourced from published references or databases and processed as described below.^4–11^

**Figure 1:**
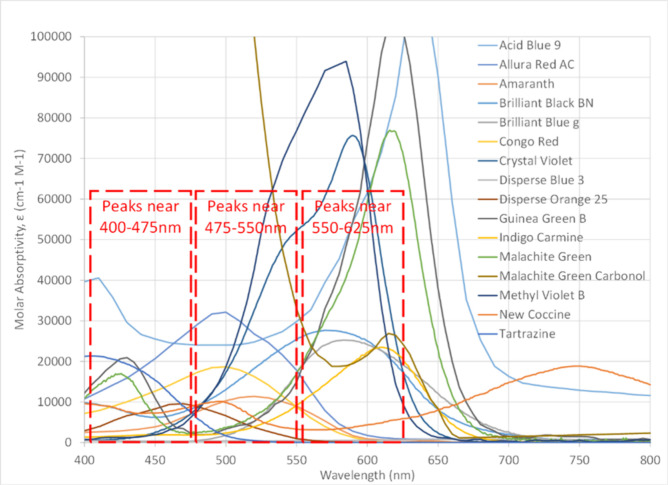
Molar absorptivity spectra of initial 16 candidate dyes.

### ‘Clean Start’ Case

As described above, we initially constrained our search to 16 dyes that we already owned, to enable later comparison between the ‘Clean Start’ and ‘Existing Supply’ approaches. Some spectral data was available as comma-separated data files (wavelength vs absorbance), permitting spreadsheet analysis. Other spectra were only available as images (e.g., from older journal articles); in these cases, numeric versions were extracted using WebPlotDigitizer, which converted (*x*, *y*) pixel coordinates into (wavelength, absorbance) values.^12^

All spectra were standardized to a common 1 nm spacing; where interpolation was necessary, a basic two-point linear method was applied. If the dye concentration was listed in the source, it was used to calculate molar absorptivity, ε (cm^–1^ M^–1^), from the absorbance. Occasionally, a single reference provided a spectrum without concentration data, but a single-wavelength measurement from another source allowed the scaling of the absorbance into molar absorptivity. High spectral fidelity was not required here, as the goal was to identify potentially useful dyes, and in later steps authentic spectra will be taken in-house and used to design the filter. These processed spectra were compiled into the ND Filter Spreadsheet, as described in the following subsection. From this point onward, the workflow was identical to that of the ‘Existing Supply’ case.

### ‘Existing Supply’ Case

If the candidate dyes are available, or were selected for purchase in the previous step, a solution of known concentration was prepared and UV–vis spectra were collected. All spectra used identical wavelength spacings (1 nm) and identical spectrometer settings.

### Design of ND Filter Spreadsheet

A flowchart summarizing our computational approach can be found in Figure 2, and the ND Filter Spreadsheet and molar absorptivities used at each stage are provided in the Supporting Information. Microsoft Excel was used here, but any software offering functionality comparable to the Solver tool can be substituted. The molar absorptivity spectra of the candidate dyes were entered into a dedicated tab (“Absorptivities”), and a second tab (“Absorbance”) was used for spectral manipulation and optimization as described below, and shown in Figure 3.

**Figure 2:**
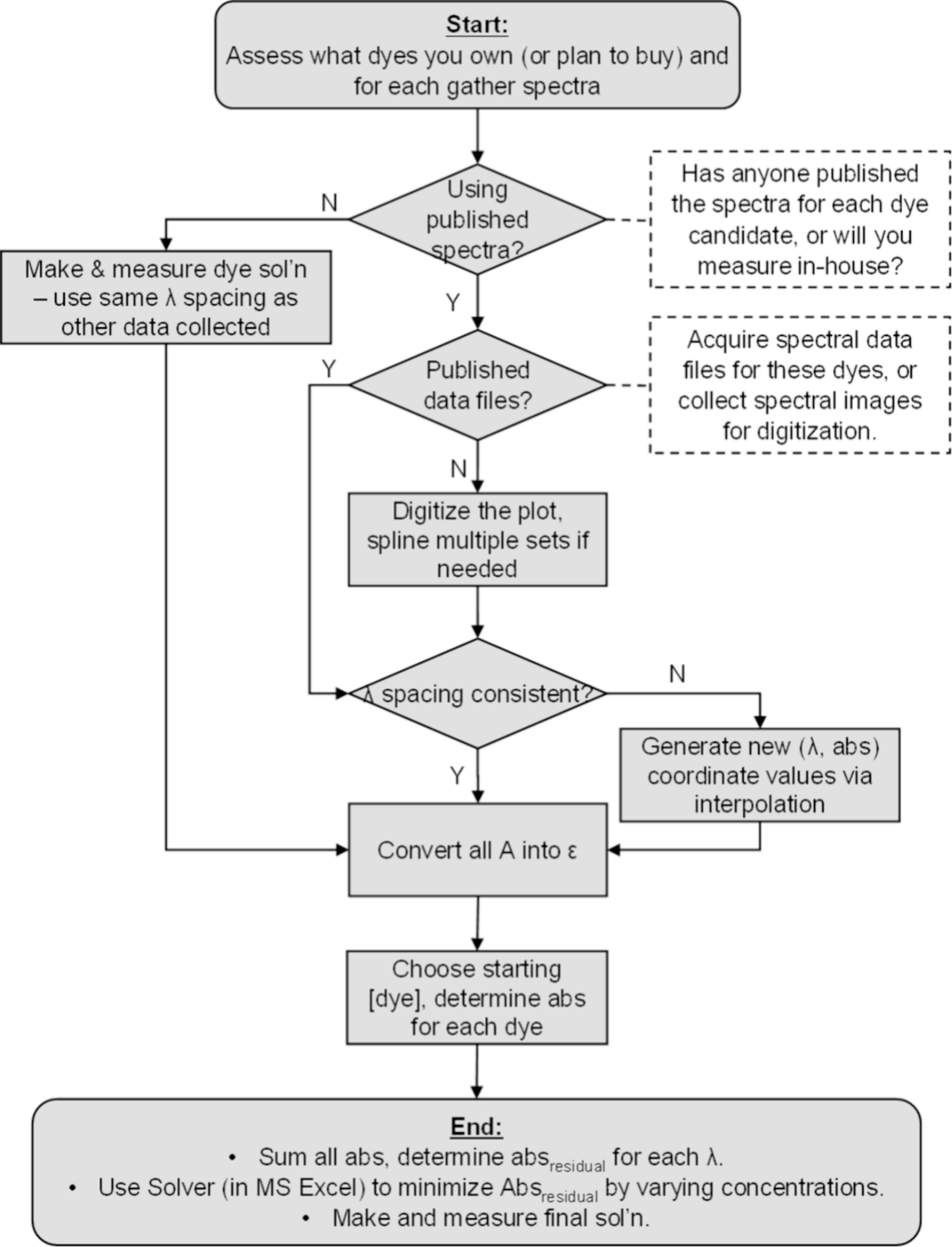
Outline of process to develop ND filter solution.

**Figure 3:**
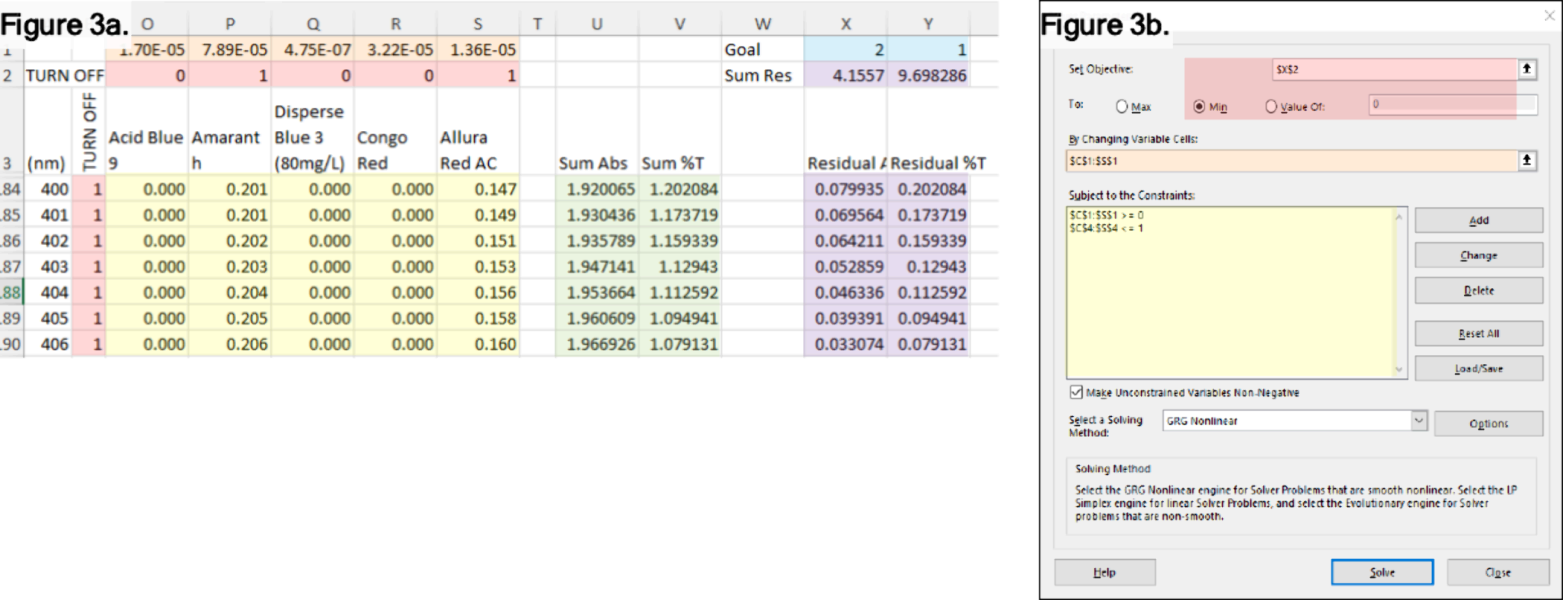
Optimization spreadsheet design and controls. (a) Layout of the optimization spreadsheet and description of elements used in calculation. Shading key. Red: toggles to add/remove a spectrum or wavelength from consideration during fitting (1 = include, 0 = exclude). Orange: these are the dye concentrations. A reasonable concentration is seeded initially, then varied by the Goal Seek function (below). Yellow: predicted abs from each dye; if either toggle is set to 0, the abs is set to 0, removing it from consideration during the Goal Seek fitting (below). Green: sum of all abs_λ_ and corresponding %T. Blue: target abs or %T. Violet: absolute value of the residual between the summed and target abs and %T, as well as the total residual for each. (b) The Goal Seek dialogue box. This spreadsheet feature is used to modify the concentrations to best fit the target abs or %T. Shading key. Red: this links to the sum of residuals (abs or %T) which is to be minimized (radio button). Orange: the concentrations; these will be varied until there is minimal residual difference between the abs or %T and the target value. Yellow: this limits the concentration to reasonable values (0–1 M).

A matrix was constructed with wavelengths in the row indices, and each dye was assigned to a separate column. The top cell of each column held the dye’s concentration (initially set to 0.000 M). To allow selective optimization for only specified wavelengths and dye subsets, without altering the core data, we implemented logic flags for both wavelength and dye inclusion (on/off switches). Only cells for which both wavelength and dye flags were set to “1” (i.e., active) were populated with calculated absorbance values; all others remained blank to prevent inclusion in the optimization process. The total absorbance for each wavelength was calculated as the sum across dye columns, producing a column of net absorbance values for all dyes selected for inclusion in the final dye mixture.

A separate input cell specified the target absorbance value for the desired ND filter. Modeled and target absorbance values were compared at each active wavelength to compute the residual error, and the sum of absolute residuals across all wavelengths was defined as the optimization metric. Excel’s Solver function was then used to minimize this total residual by varying the dye concentration cells; output concentrations were monitored to verify physically realistic and useful concentrations were output. Dyes contributing negligibly to the total absorbance spectrum were identified by their low optimized concentrations (e.g., 1.00 × 10^–9^ M), and the effect of removing that dye via the logic flag was observed (in case some molar absorptivities were large enough that even a small concentration impacted the overall spectrum). After removing dyes from consideration, the optimization was rerun. This enabled rapid identification of a core dye set, and corresponding target concentrations for solution preparation.

### Generation of the ND Solution

As discussed above, in the ‘clean start’ case the spectral data may have limited quality due to import artifacts; at this stage, the key dyes identified from the spreadsheet optimization process can be acquired, and more refined spectral data can be taken and added to the spreadsheet. Similarly, for the ‘Existing Supply’ case, the spreadsheet will have identified a narrower set of candidate dyes. In both cases, we proceeded using in-house spectral data; any chemical impurities will now be explicitly accounted for in this actual spectral data. At this point, the spreadsheet model suggested 6 dyes for the ND filter set; however, while preparing samples of buffered dyes for spectral analysis we found that one of the candidate dyes (Disperse Orange 25) had poor solubility. As a result, removed that dye from the optimization process (via the ‘off’ flag), and reinitialized the optimization process including all other dyes. Dyes with minimal contribution (concentrations to the absorbance profile were iteratively removed, leaving 6 dyes out of the 16 in the final spreadsheet optimization.

The spreadsheet optimization process yields the required concentration for each of the 6 dyes in the solution; we prepared single-component stock solutions for each dye, ensuring that no pH dependent changes in spectra would occur (pH 7 0.01 M phosphate buffer: KH_2_PO_4_, Fisher Scientific and K_2_HPO_4_, Sigma-Aldrich).

The mass of each of the 6 dyes (Indigo Carmine, Tartrazine, Brilliant Blue G, Acid Blue 9, Congo Red, and Amaranth) needed was added to a 100.00 mL volumetric flask and diluted with to create the final ND Filter solution.

The absorbance spectrum for this ND filter solution was measured and compared to the modeled spectrum.

### Photostability of the ND Filter Solution

To validate that the ND filter solution was photostable for most uses, an aliquot of the ND filter solution was placed into a cuvette and exposed to repeated laser pulses from an Opolette 355 LD class IV OPO-laser system (peak pulse energy 5.1 mJ, pulse length 5 ns, 2.9 W/cm). The laser was scanned at 1 nm intervals from 410 to 650 nm, with 98 shots fired at each wavelength at a 20 Hz repetition rate, and at full power (23,520 laser shots total). The UV–vis spectrum was recorded and compared to the original spectrum to detect any spectral changes.

## Results and Discussion

### ND Filter Solution

The spreadsheet optimization process identified six dyes for the ND filter mixture. Table 1 summarizes the dyes and their costs to produce an ND filter stock solution. 100 mL of ND filter solution with abs = 2 would cost $0.54 to make if the chemicals had been purchased exclusively for this purpose. This stock can readily be diluted and stored in sealable cuvettes to generate an ND filter attenuator series.

**Table 1:** Composition of 100 mL ND Filter Solution (Costs in USD, July 2025)

	Acid Blue 9	Amaranth	Brilliant Blue g	Congo Red	Indigo Carmine	Tartrazine		
mass (g)	25	100	10	10	25	25		
price	$47.40	$58.60	$52.60	$12.85	$53.70	$19.60		
source	Alfa Aesar	Sigma-Aldrich	Alfa Aesar	Flynn Scientific	Sigma	Alfa Aesar		
price (g)	$1.90	$0.59	$5.26	$1.29	$2.15	$0.78		
amt used (g)	0.0108	0.1070	0.0629	0.0179	0.0385	0.0314		
cost used	$0.020	$0.063	$0.331	$0.023	$0.083	$0.025	total cost	$0.544

We compared the modeled absorbance spectrum against the experimentally measured spectrum of the stock solution (Figure 4). At this stage, all spectra data used are experimentally measured, ensuring that spectra of the dyes as owned and measured (impurities in the dye, spectrometer characteristics, etc.) were used in the optimization process. The solution exhibited a flat absorbance profile across the optimized 425–575 nm wavelength range. The observed absorbance ranged from 2.04 to 2.16 across 425–575 nm (0.7–0.9%T), with a mean of 2.09 and a standard deviation of 0.03. This closely matched the target absorbance of 2.00 from our modeling process.

**Figure 4:**
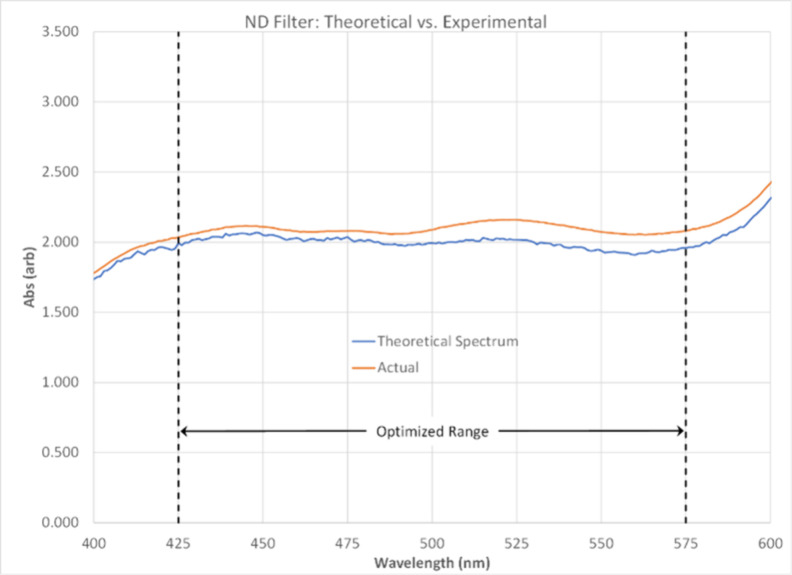
Modeled vs actual ND filter solution spectra.

The slight deviation between the modeled and experimental spectra is likely due to uncertainty in the calculated molar absorptivities. In order to make a useful absorbance ND filter, the stock solutions must be higher than the target absorbance, generally placing the absorbance of the stock solution outside the range of the spectrometer. This requires a stock solution to be diluted for spectral measurement. Any dilution error will couple with any uncertainty caused in the spectral measurement (fluctuations in measured absorbance, the higher percent error for small absorbance values along the sides of absorbance peaks, etc.). These effects may be minimized by optimizing spectral quality (slow scan rates, careful baseline monitoring, etc.). Additionally, an additional dye can be added with an absorbance peak just outside the optimized wavelength range to ensure flatness near the edge of the range.

### Filter Solution Robustness

To evaluate the photostability of our ND filter solution, we exposed it to harsher conditions than are typical to assess the photostability of the dye mixture (OPO laser as described above, 23,520 laser shots, 20 Hz repetition rate, maximum output power, no solution mixing). Figure 5 presents the pre- and postirradiation spectra, with the differential spectrum magnified in blue. The spectra were visually indistinguishable, with Δabs decreases ranging from 0.001 to 0.010 (avg = 0.004).

**Figure 5:**
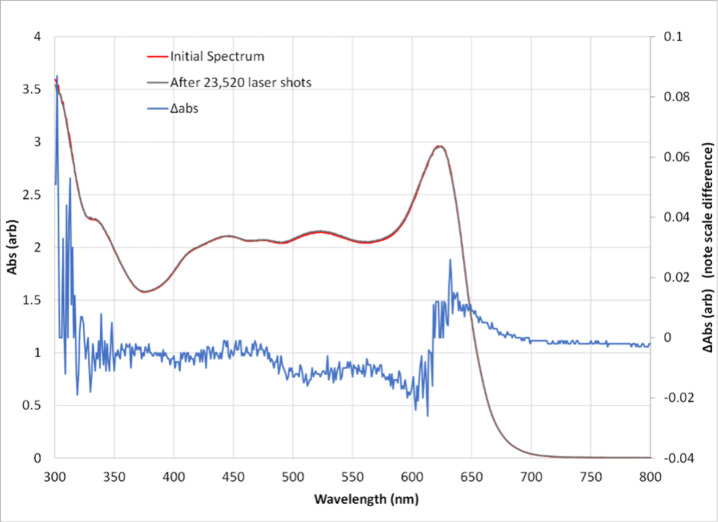
Robustness of ND filter solution. The spectra before and after 23,520 laser shots show minimal difference; the differential spectrum is shown in blue and magnified for visibility.

Researchers working at higher powers, shorter pulse widths, or high pulse frequencies should use a similar approach to verify a lack of photodegradation for their unique dye mixture and use case, and could further mitigate photobleaching via mixing within the cuvette, or pumping the attenuation solution through a flowthrough cuvette. At higher powers or with focused beams, users may need to monitor for thermal lensing effects stemming from the use of an absorptive attenuation filter. In our experiments, no beam deflection was observed indicating that the ND filter solution did not exhibit observable beam deflection, indicating a lack of thermal lensing effects under our conditions. If a focused beam or a more intense light source is being attenuated, users should validate that the beam position remains stable in their application.

### Turning Difficulties into Opportunities

Recent disruptions, from the COVID-19 pandemic to hurricane-related laboratory closures, have highlighted the need for flexible approaches that support educationally meaningful student engagement. While lecture courses can often pivot to online formats with relative ease, research experiences pose a greater challenge. These events underscore the importance of proactively designing remote-compatible research strategies that preserve both educational value and scientific progress.

In our case, communication proved essential. Regular video meetings allowed our research group to maintain momentum, discuss available data, and plan for future in-person work. We preserved the traditional structure of a chemistry research group, where student projects contribute to the principal investigator’s broader research goals, and theoretical work is directed toward practical outcomes.

This led us to pursue the development of a custom ND filter solution using dyes already in our inventory. However, without access to the lab or instrumentation, we initially operated as if starting from scratch (the “clean start” scenario). Students engaged in data mining, spectral digitization, and spreadsheet modeling, developing foundational research skills in the process. Their early modeling efforts, based on published spectra, closely matched the final experimental results, validating the utility of the clean start approach.

This experience demonstrated the value of integrating computational and literature-based tasks into undergraduate research, especially when laboratory access is limited. The workflow bridged theoretical understanding and experimental design, showing that meaningful scientific contributions can emerge from pedagogically motivated constraints. The success of this hybrid model suggests its broader applicability in course-based research experiences and student training programs.

## Conclusions

We have demonstrated a generalizable approach for users to easily and inexpensively generate customizable, low-cost optical attenuators using either already-owned dyes or via targeted and optimized purchases. The resulting liquid ND filters demonstrate spectral flatness on par with commercially available filters at a fraction of the cost. Especially when multiple attenuation levels are used. The dye set demonstrated here showed robustness to photodegradation, and no thermal lensing was observed under our conditions. This spreadsheet-based modeling and optimization approach is broadly generalizable and allows users to tailor their own wavelength ranges.

This work also sits at the intersection of mentored student research and undergraduate education. During the COVID-19 pandemic, for a time it was difficult to meet with students in the lab. This work required a strategy that started students in research outside the laboratory, with this work leading directly into actionable laboratory tasks and outcomes. This strategy can be scaled up and used in course-based research experiences or normal research student training, with literature-based and hands-on research exposure into spectroscopy, data processing, and experimental design in undergraduate settings. Finally, we present an approach to develop ND filters in education or research at minimal cost.

## Supplementary Material





## Notes

Lot numbers for Indigo Carmine, Tartrazine, Brilliant Blue G, Acid Blue 9, Congo Red, and Amaranth; MS excel file used for ND filter optimization (PDF)
ND filter spreadsheet (XLSX)

## ‡

Mercer University, College of Pharmacy, 3001 Mercer University Dr, Atlanta, Georgia 30341, United States

## §

University of Central Florida, College of Medicine, 4000 Central Florida Blvd, Orlando, Florida 32816, United States.

## ∥

University of Florida, College of Pharmacy, 1225 Center Dr, Gainesville, Florida 32610, United States.
