# Supplementary material for: Generation of Low-Cost User-Customizable Neutral Density Filters and the Involvement of Undergraduate Researchers
Source: ACS Omega. 2025 Dec 19;11(1):826–32. doi: 10.1021/acsomega.5c07533 (PMC12809282; doi:10.1021/acsomega.5c07533)
Supplement: Supplementary file 1 [file ao5c07533_si_001.pdf]

Generation of Low-Cost User-Customizable Neutral Density Filters, and the Involvement of Undergraduate Researchers

Julia Filip‡, Joseph Eppich‡, Melissa Y. Gallardo‡, Andrew S. Hamilton‡, Noelle Vermost‡, Kevin W. Davies*

Department of Chemistry and Physics, Florida Gulf Coast University, 10501 FGCU Blvd South, Fort Myers, FL 33965-6565, United States

*Corresponding Author. Fax: 239-590-7200. Email: kdavies@fgcu.edu

‡J. Filip, J. Eppich, M. Y. Gallardo, A. S. Hamilton, and N. Vermost contributed equally to this work.

Supporting Information:

Spectral data for the dyes used are provided in the accompanying spreadsheet. Provided below are details about the dyes used in the final ND filter solution.

Indigo Carmine, lot BCBC4486V
Tartrazine, Sigma Aldrich lot 116H1443
Brilliant Blue G, Alfa Aesar lot N07G028
Acid Blue 9, lot 126H3473
Congo Red, Sigma Aldrich lot 49H2608
Amaranth, Fluka lot 414965
